# Study on the Function of Leptin Nutrient Acquisition and Energy Metabolism of Zebrafish (*Danio rerio*)

**DOI:** 10.3390/ijms252111647

**Published:** 2024-10-30

**Authors:** Jiaqi Wu, Wuyuan Zhuang, Ke Lu, Lixin Zhang, Yuye Wang, Farui Chai, Xu-Fang Liang

**Affiliations:** 1 College of Fisheries, Chinese Perch Research Center, Huazhong Agricultural University, Wuhan 430070, China; 2021308010016@webmail.hzau.edu.cn (J.W.); wuyuanzhuang_1996@163.com (W.Z.); luke@mail.hzau.edu.cn (K.L.); zlx8886@163.com (L.Z.); wangyuye@webmail.hzau.edu.cn (Y.W.); chaifarui@163.com (F.C.); 2 Engineering Research Center of Green development for Conventional Aquatic Biological Industry in the Yangtze River Economic Belt, Ministry of Education, Wuhan 430070, China

**Keywords:** *leptin*, glycometabolism, lipid metabolism, zebrafish

## Abstract

Leptin plays an indispensable role in energy homeostasis, and its involvement in metabolic activities has been extensively explored in fish. We generated mutant lines of *leptina* (−5 bp) and *leptinb* (+8 bp) in zebrafish using CRISPR/Cas9 technology to explore the metabolic characteristics of *lepa* and *lepb* mutant zebrafish in response to high glucose nutritional stress induced by high levels of carbohydrates. The results were as follows: the body weight and food intake of adult zebrafish of the two mutant species were increased; the visceral fat accumulation, whole-body crude lipid, and crude protein contents of *lepb*^−/−^ were increased; and the visceral fat accumulation and crude lipid in *lepa*^−/−^ zebrafish were decreased. The blood glucose levels of the two mutant zebrafish were increased, the mRNA expression levels of glycolytic genes *pk* and *gck* were decreased in the two mutant zebrafish, and there were differences between *lepa*^−/−^ and *lepb*^−/−^ zebrafish. The expressions of glycogen synthesis and decomposition genes were inhibited and promoted, respectively. The expression of adipose synthesis genes in the liver and muscle was stimulated in *lepb*^−/−^ zebrafish but suppressed in *lepa*^−/−^ zebrafish. Lipolysis and oxidation genes were also stimulated in *lepa*^−/−^ zebrafish livers, while the livers of *lepb*^−/−^ zebrafish were stimulated but muscle was inhibited. In conclusion, the results indicate that *lepa* plays a major role in glucose metabolism, which is conducive to promoting glucose utilization and lipogenesis, while *lepb* mainly promotes lipolysis and oxidation, regulates protein generation, and plays a minor role in glucose metabolism.

## 1. Introduction

As an important source of protein, the demand for fish is increasing. In order to increase fish production, improvement in feed utilization has become critical. Therefore, adjusting the composition of nutrients in feed is the key to improving feed utilization. However, fish feed with carbohydrate and fat levels at or above the upper limit usually trigger diseases such as diabetes or obesity, seriously affecting the utilization of feed by fish and, thus, yields. Obesity and type 2 diabetes mellitus (T2DM) are also among the most commonly encountered and difficult-to-cure health problems in humans today, and the World Health Organization (WHO) has declared both diseases to be global epidemics [1]. A key feature of these conditions is disrupted leptin signaling. Leptin is a hormone that is formed as a result of spontaneous mutations in the autosomal obesity gene (Ob) and plays a key role in energy homeostasis, as described by Zhang et al. (1994) [2].

Leptin consists of 167 amino acids and has a protein size of 16 kDa [3]. The sequence similarity of leptin between bony fish and mammals ranges from approximately 13% to 25% [4,5,6,7]. There is only one isoform of *leptin* and its receptor in mammals, but at least two paralogous homologs of *leptin* and its receptor have been found in fish [8]. Fish-specific whole-genome duplication (WGD) events lead to the multicopy generation of scleractinian genes [9]. In mammals, adipose tissue and the brain are generally recognized as major sites of leptin synthesis and receptor expression [10,11,12]. In bony fish, *leptin* is usually expressed in the liver, and the levels of *leptin* expression in adipose tissue differ depending on the fish species [13,14,15,16,17,18,19]. The expression patterns of the two isoforms of leptin also differ, with *leptina* (*lepa*) being predominantly expressed in the liver and *leptinb* (*lepb*) expression levels higher in the ovaries [4,15]. The specificity of tissue expression of *leptin* indicates that its biological function may also be specific.

In mammals, changes in *leptin* levels mainly affect food intake and energy expenditure [20]. Food intake levels are associated with changes in hypothalamic neuropeptide expression. In mammals, leptin plays an anorexic role by inhibiting the expression of neuropeptide Y (NPY) and agouti-related protein (AgRP) and stimulating the expression of pro-opiomelanocortin (POMC) and cocaine and amphetamine-regulated transcription (CART) [21,22,23]. Leptin also plays a role in feeding by acting on receptors synthesized in the hypothalamus of fish, influencing appetite factors [24].

The homeostasis of energy, i.e., the metabolism maintained by animals, is the basis of normal life activities, and the imbalance of homeostasis can lead to obesity or diabetes [25]. Feeding and energy metabolism in mammals can be regulated by neurons with glucose-sensing properties, which are distributed in the hypothalamus [26,27]. Leptin promotes energy homeostasis by regulating lipid and carbohydrate metabolism in peripheral tissues [28]. Leptin also mobilizes lipids for energy use by promoting lipolysis, inhibiting lipogenesis, and up-regulating fatty acid oxidation in the liver, muscle, and adipose tissue [29,30,31,32]. Dysfunctional metabolism; decreased body temperature and oxygen consumption; accumulation of lipids in tissues; and the development of insulin resistance, leading to elevated plasma glucose levels, have been reported in leptin-deficient obese mice [33].

Although leptin is well known in mammals for its adipose-inhibitory effects, it also regulates glucose homeostasis—an effect that is independent of its influence on adiposity. To date, the mechanisms by which leptin exerts its glucoregulatory effects remain largely unknown, especially in fish, and the effect on fat is also ambiguous. There are two distinct isoforms of leptin in bony fish, and we hypothesize that some of the functions of the two isoforms may overlap with those of mammals but that a particular isoform may play its own unique role. In order to study the function of the leptin gene, the CRISPR/Cas9 system was utilized to mutate the leptin gene in zebrafish. Zebrafish with a loss of function of the leptin gene were used as experimental subjects to investigate the differences in feeding, energy metabolism, and other aspects related to the leptin gene in zebrafish and to determine the role of leptin in their feeding and metabolism. This research also aimed to analyze the functional differentiation of the *lepa* and *lepb* genes in zebrafish and to provide effective evidence for the study of the different physiological roles exerted by leptin in fish. It further aims to improve the understanding of the role of leptin in non-mammals and is expected to provide theoretical references for solving the obesity and diabetes problems currently faced by human beings.

## 2. Results

### 2.1. Construction of Leptin Mutant in Zebrafish

**Figure 1:**
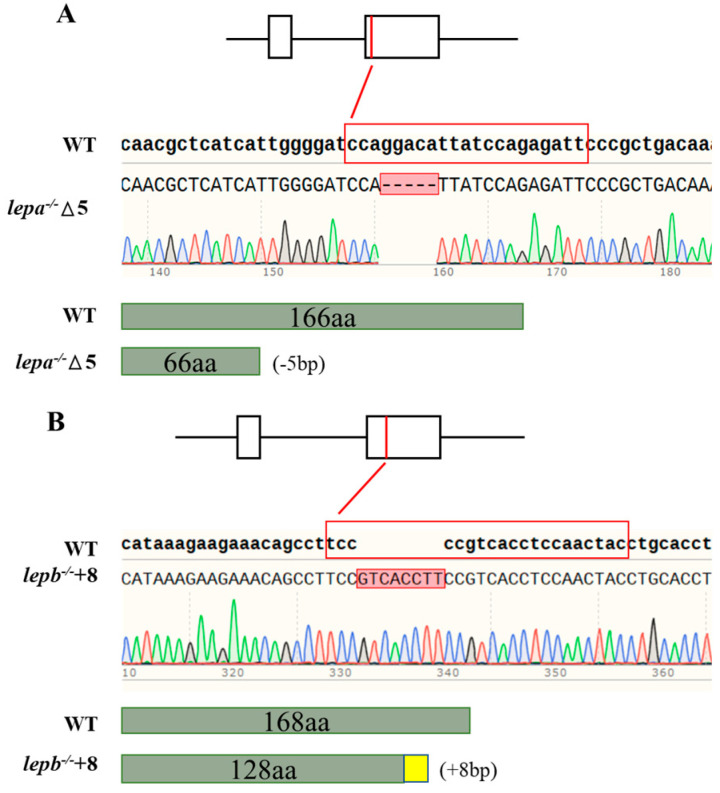
(**A**,**B**) The sequence result of mutant genes. Dotted lines represent missing bases, and red squares represent inserted bases. The sgRNA sequences are highlighted in red, and the −5 bp and +8 bp deletions are indicated by sequencing validation.

In this study, mutant *lepa*^−/−^ and *lepb*^−/−^ zebrafish strains were obtained. The *lepa*^−/−^ mutant lacks five bases, while the *lepb*^−/−^ mutant adds eight bases (Figure 1). By comparing the gene sequences and predicted protein structures with wild-type samples, it was found that the *lepa*^−/−^ (−5 bp) mutant had a translation termination at 67 amino acids, while the *lepb*^−/−^ (+8 bp) mutant had a translation termination at 154 amino acids. All of these mutants have deletions in their protein domains.

### 2.2. Analysis of Growth Performance of Lepa^−/−^ and Lepb^−/−^ Mutants Under High-Glucose Diet Induction

After 60 days of high-glucose diet induction, it was found that compared with wild-type zebrafish (Table 1), the body weight of *lepa*^−/−^ and *lepb*^−/−^ zebrafish increased by 137.18% (*p* < 0.05) and 135.51% (*p* < 0.05), respectively (Table 1). In terms of the entero–lipid ratio, that of the *lepa*^−/−^ zebrafish ratio was significantly decreased (*p* < 0.05), while that of the *lepb*^−/−^ zebrafish was distinctly increased (*p* < 0.05).

Further observation of the changes in body components of *lepa*^−/−^ and *lepb*^−/−^ zebrafish showed that there was a tendency for an elevated crude protein content in *lepa*^−/−^ zebrafish with no statistically significant differences and a significant elevation in *lepb*^−/−^ zebrafish (*p* < 0.05) compared to the control group. The data indicated that the crude fat content of *lepa*^−/−^ zebrafish was prominently reduced (*p* < 0.05), while the visceral fat deposition of *lepb*^−/−^ zebrafish was elevated (*p* < 0.05).

### 2.3. Gene Expression Related to Appetite and Food Intake

**Figure 2:**
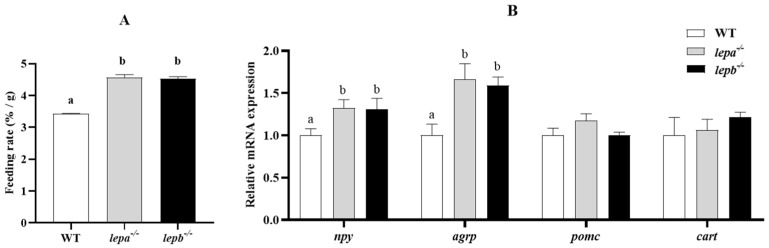
Genee expression related to appetite and food intake of *lepa*^−/−^, *lepb*^−/−^, and wild-type zebrafish. (**A**) Food intake of *lepa*^−/−^, *lepb*^−/−^, and wild-type zebrafish. (**B**) Expression levels of appetite-related genes in the brain of the three genotypes of zebrafish. Different letters indicate significant differences (*p* < 0.05).

After high-glucose induction, the weight-gain rate of the mutants was significantly higher than that of the wild type, which may be due to abnormal leptin levels in the mutants, blocked appetite-inhibiting signals, or stimulated appetite-promoting signals. Therefore, we tested the feeding rates of the three genotypes of zebrafish, and the results indicated that the feeding rates of *lepa*^−/−^ and *lepb*^−/−^ zebrafish were significantly higher than those of wild-type zebrafish (*p* < 0.05) (Figure 2A). The mRNA expression levels of *agrp* and *npy* in *lepa*^−/−^ and *lepb*^−/−^ zebrafish were significantly elevated compared to those of wild-type zebrafish (*p* < 0.05) (Figure 2B), while the mRNA expression levels of appetite-suppressing genes *pomc* and *cart* in *lepa*^−/−^ and *lepb*^−/−^ zebrafish were not significantly different from those in wild-type zebrafish (*p* > 0.05).

### 2.4. Changes of Blood Physiological and Biochemical Related Indicators in Lepa^−/−^ and Lepb^−/−^ Zebrafish

**Figure 3:**
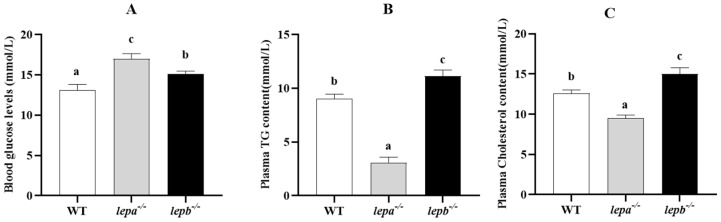
The effect of leptin on blood glucose and lipid levels. (**A**) Blood glucose levels, (**B**) plasma TG levels, and (**C**) plasma cholesterol levels in the three genotypes of zebrafish. Different letters indicate significant differences (*p* < 0.05), with *n* = 6 for each genotype.

We further explored the impact of leptin on blood glucose and lipid levels, as shown in Figure 3. The blood glucose levels of the mutant zebrafish were obviously higher than those of the wild-type zebrafish (*p* < 0.05), and the blood glucose levels of *lepa*^−/−^ zebrafish were higher than those of *lepb*^−/−^ zebrafish (*p* < 0.05) (Figure 3A). Compared with wild-type zebrafish, the levels of triglycerides (TG) and cholesterol in the blood of *lepa*^−/−^ zebrafish were significantly reduced (*p* < 0.05), while those in *lepb*^−/−^ zebrafish were significantly increased (*p* < 0.05) (Figure 3B,C).

### 2.5. Lepa^−/−^ and Lepb^−/−^ Zebrafish Liver and Muscle Glycogen and Fat Contents

**Figure 4:**
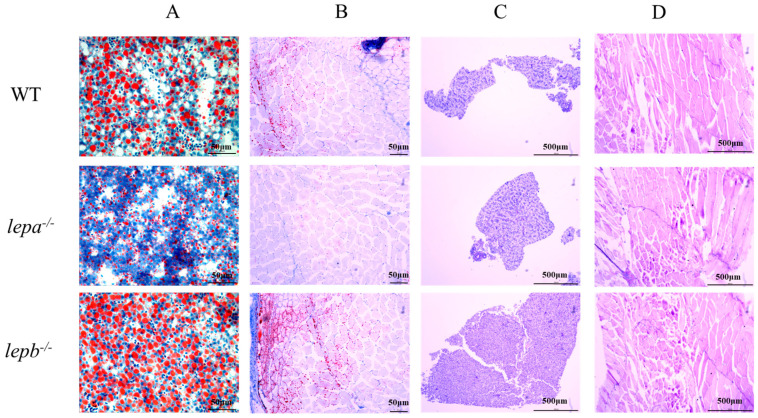
Oil red O and AB-PAS staining of liver and muscle tissue of three genotypes zebrafish. (**A**) Oil red O staining of the liver. (**B**) Oil red O staining of muscle. (**C**) AB-PAS staining of the liver. (**D**) AB-PAS staining of muscle.

The glycogen and fat contents in the liver and muscle tissues of *lepa*^−/−^, *lepb*^−/−^, and wild-type zebrafish were detected by staining with Oil Red O and AB-PAS. The results of oil red O staining showed that the fat content of *lepa*^−/−^ zebrafish was lower, while that of *lepb*^−/−^ zebrafish was higher in muscle and liver compared to the wild-type fish (Figure 4A,B). AB-PAS staining revealed no significant differences in glycogen content among the three genotypes in liver and muscle tissues (Figure 4C,D).

**Figure 5:**
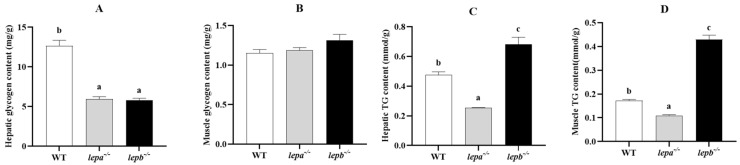
The levels of glycogen and triglyceride (TG) in the liver and muscle of three genotypes of zebrafish. (**A**) Glycogen levels in the liver. (**B**) Glycogen levels in muscle. (**C**) TG levels in the liver. (**D**) TG levels in muscle. The letters a, b, and c in the bar chart represent significant differences for each index among *lepa*^−/−^, *lepb*^−/−^, and wild-type zebrafish (*p* < 0.05), with *n* = 6 for each genotype.

The contents of glycogen and TG in the muscle and liver of zebrafish were detected by a liver/muscle glycogen assay kit and TG assay kit. The glycogen contents of *lepa*^−/−^ and *lepb*^−/−^ zebrafish in the liver were significantly lower than those of wild-type zebrafish (*p* < 0.05) (Figure 5A). In muscle tissue, there was no significant difference in glycogen content among *lepa*^−/−^, *lepb*^−/−^, and wild-type zebrafish (*p* > 0.05) (Figure 5B). Compared to the TG contents of wild-type zebrafish, the TG contents of *lepa*^−/−^ zebrafish in both muscle and the liver were significantly decreased (*p* < 0.05), while the TG contents of *lepb*^−/−^ zebrafish were significantly increased (*p* < 0.05) (Figure 5C,D).

### 2.6. Expression Analysis of Genes Related to Glucose and Lipid Metabolism

**Figure 6:**
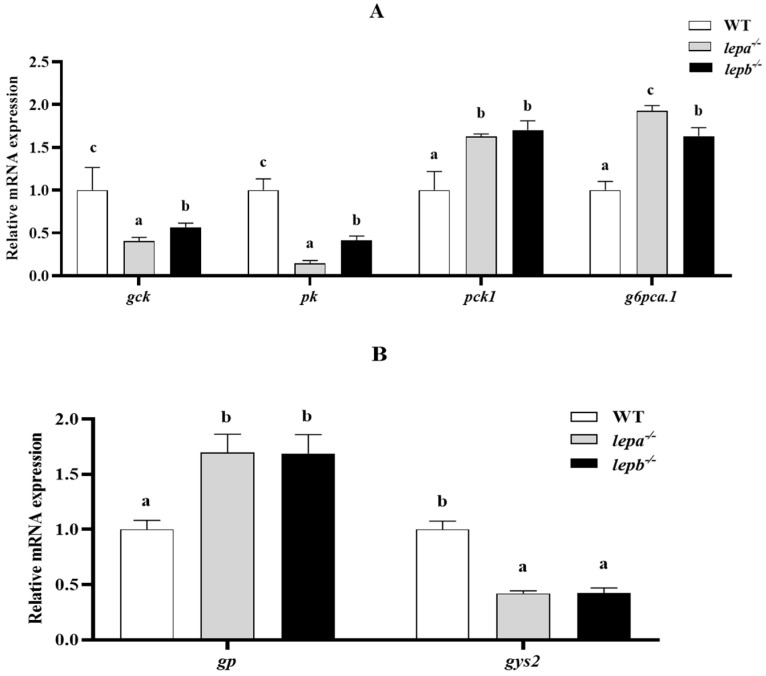
Expression levels of genes related to glucose metabolism in the liver. (**A**) Glycolysis and gluconeogenesis genes. (**B**) Glycogen metabolism gene. The letters a, b, and c in the bar chart represent significant differences foreach index among *lepa*^−/−^, *lepb*^−/−^, and wild-type zebrafish (*p* < 0.05), with *n* = 6 for each genotype.

We further examined genetic changes related to lipid metabolism, glucose metabolism, and protein synthesis. Compared with the wild-type zebrafish, the mRNA expression levels of glycolysis genes *glucokinase* (*gck*) and *pyruvate kinase* (*pk*) in the mutants were significantly decreased (*p* < 0.05) (Figure 6A). The expressions of gluconeogenesis genes *phosphoenolpyruvate carboxykinase 1* (*pck1*) and *glucose-6-phosphatase a catalytic subunit tandem duplicate 1* (*g6pca.1*) were significantly increased (*p* < 0.05) (Figure 6A). The expression of glycogen phosphorylase gene *glycogen phosphorylase* (*gp*) was significantly higher (*p* < 0.05), and that of *glycogen synthase 2* (*gys2*) was significantly lower (*p* < 0.05) (Figure 6B). These findings are consistent with the results of glycogen content assays conducted in liver tissue.

**Figure 7:**
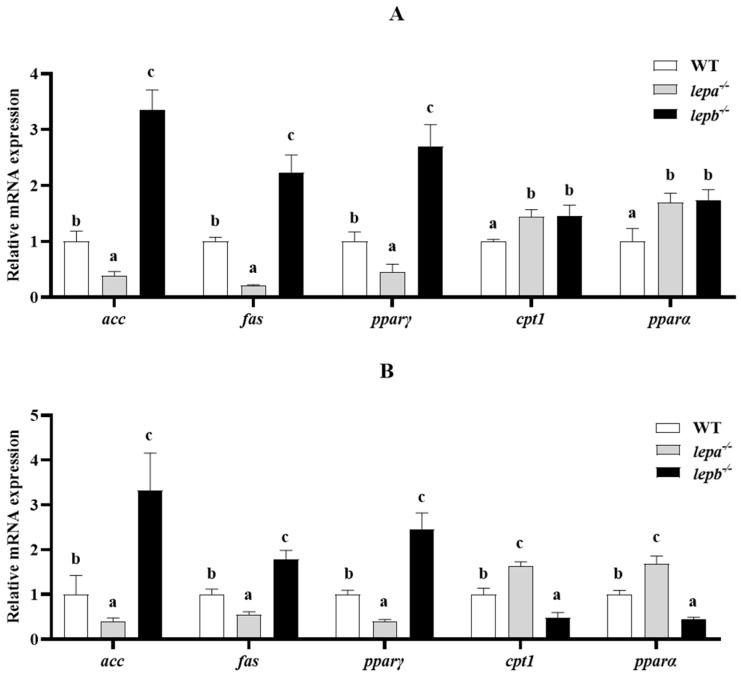
Expression levels of lipid metabolism genes in the liver (**A**) and muscle (**B**) of three genotypes of zebrafish. The letters a, b, and c in the bar chart represent significant differences for each index among *lepa*^−/−^, *lepb*^−/−^, and wild-type zebrafish (*p* < 0.05), with *n* = 6 for each genotype.

In the assay of lipid metabolism-related genes, compared to wild-type zebrafish, *acetyl-CoA carboxylase alpha* (*acc*), *fatty acid synthase* (*fas*), and *peroxisome proliferator-activated receptor gamma* (*pparγ*) contents were significantly elevated in *lepb*^−/−^ zebrafish liver and muscle tissues (*p* < 0.05), while they were significantly lower in *lepa*^−/−^ zebrafish liver and muscle tissues (*p* < 0.05) (Figure 7). *Carnitine palmitoyl transferase 1B* (*cpt1b*) was significantly increased (*p* < 0.05) in both the liver and muscle of *lepa*^−/−^ zebrafish, whereas the results for *peroxisome proliferator-activated receptor alpha* (*pparα*), a key transcription factor in fat oxidation, followed the same trend as *cpt1b* in *lepa*^−/−^ zebrafish liver and muscle tissues. Interestingly, *cpt1b* was significantly increased (*p* < 0.05) in the liver of *lepb*^−/−^ zebrafish but significantly decreased (*p* < 0.05) in muscle.

## 3. Discussion

Leptin plays an important role in controlling food intake and growth energy metabolism in mammals, and little research has been performed on this function in fish or in non-mammals in general [34]. In this experiment, we explored the role of leptin in nutrient acquisition and energy allocation using pure *lepa*^−/−^ and *lepb*^−/−^ zebrafish mutants constructed by CRISPR/Cas9 gene editing.

Previous studies have shown that leptin in fish such as goldfish [35], rainbow trout [14,36], and Mandarin fish [37] is involved in the regulation of body feeding and body weight. Both NPY and AgRP are potent appetite stimulants. Their expression levels in the hypothalamus have been extensively demonstrated to be inhibited by leptin in mammals [34,38,39,40,41,42,43,44], and their appetite-stimulating effects have been studied in zebrafish [45,46,47]. Consistent with these observations, *lepa*^−/−^ and *lepb*^−/−^ zebrafish showed significantly higher rates of body weight gain and food intake than wild-type zebrafish following a high-glucose diet. Elevated feeding in *lepa*^−/−^ and *lepb*^−/−^ zebrafish and significantly higher levels of appetitive *npy* and *agrp* mRNA in *lepa*^−/−^ and *lepa*^−/−^ zebrafish than in wild-type zebrafish suggest that up-regulation of appetitive genes in the mutants may enhance their feeding.

Fewer studies have been conducted to explore whether *lepa* and *lepb* differ in glucose metabolism in fish. In the present study, *lepa* and *lepb* mutants were fed a high-glucose diet and found to have elevated blood glucose levels in both mutant fish, with *lepa*^−/−^ zebrafish having higher blood glucose levels than *lepb*^−/−^ zebrafish. We also observed changes in glycogen content by AB-PAS staining of zebrafish liver and muscle tissues; both mutant zebrafish had reduced liver glycogen contents and no changes in muscle tissue glycogen compared to the wild-type zebrafish, similar to the results obtained with leptin treatment in goldfish [35]. The expression of glycolysis-related genes *pk* and *gck*, as well as gluconeogenesis-related genes *pck1* and *g6pca*, was reduced in the livers of both mutants compared to the wild-type zebrafish. One of the most important findings in our study was that *lepa*^−/−^ zebrafish were more affected than *lepb*^−/−^ zebrafish by the processes of glycolysis and gluconeogenesis. This also explains the higher blood glucose levels in both mutant zebrafish compared to the wild-type zebrafish, as well as the higher blood glucose levels in *lepa*^−/−^ zebrafish compared to *lepb^−^^/^^−^* zebrafish—a difference that implies primary and secondary roles for *lepa* and *lepb* in metabolic glucose utilization. This finding is consistent with the results reported by Londraville et al., who showed that leptin regulates the expression levels of *pk* and *pklr*, modulating glycolysis and thereby maintaining glucose homeostasis in the body [48,49]. Interestingly, for the first time in zebrafish leptin research, we found functional differences between *lepa* and *lepb* in terms of their involvement in glycolysis to maintain glucose homeostasis. *Lepa* may play a primary role in the maintenance of organismal glucose homeostasis by participating in glycolysis and gluconeogenesis, whereas *lepb* may play a secondary role in the maintenance of glucose homeostasis by participating in organismal glucose metabolism.

The liver plays an important role in the body’s energy metabolism, and adipose tissue plays a key role in energy storage [50]. When the amount of fat exceeds the liver’s ability to metabolize it, fat accumulates in the liver [51]. In mammals, fat deposition is associated with leptin, which mainly promotes fat hydrolysis and inhibits fat deposition, thereby maintaining energy metabolism and preventing obesity [52,53,54,55]. Intensive research on fish leptin in recent years has revealed that it is involved in processes related to lipid metabolism [56,57]. In this study, the biological functions of *lepa* and *lepb* in lipid metabolism induced by a high-carbohydrate diet were explored in zebrafish. The results revealed that triglyceride and cholesterol levels were reduced in *lepa*^−/−^ zebrafish, either in blood or in liver and muscle tissues, while the opposite was true for *lepb^−/^^−^* zebrafish. These findings support the notion that leptin is involved in the process of lipid metabolism induced by a high-glucose diet. In molecular-level analyses of liver and muscle tissues, the expression levels of genes involved in adipogenesis, such as *fas*, *acc*, and *foxo1a*, were all significantly up-regulated in *lepb^−/^^−^* zebrafish, whereas *fas* and *acc* expression levels were significantly decreased in *lepa*^−/−^ zebrafish. In combination with the above results, *lepb^−/^^−^* zebrafish also had high levels of body fat and fat deposition, while *lepa*^−/−^ zebrafish had the lowest such levels. Our data suggest that liposynthesis is stimulated in *lepb^−/^^−^* zebrafish, while it is impaired in *lepa*^−/−^ zebrafish. Combined with the process of glucose metabolism, it can be deduced that the conversion of glucose to lipids is blocked in zebrafish in the absence of *lepa*, whereas in the absence of *lepb* in zebrafish, lipids are accumulated due to the failure of lipolytic metabolism.

The expression of *cpt1b*, a sublipolysis-associated enzyme in the liver, was up-regulated in both mutant zebrafish but differed in liver and muscle tissues, possibly due to the negative feedback regulation of the organism caused by the higher liver fat content in *lepb*^−/−^ zebrafish. This leads to an enhanced capacity for organismal fat oxidation. Similarly, different results were seen after *leptin* or receptor knockout in zebrafish [58,59] and medaka [18]. Phenotypic leptin or receptor knockout differences may be due to mutations in different alleles [60]. Induced by a high-glucose diet, our data support the hypothesis that the effect of leptin on lipid metabolism is highly conserved throughout the phylogeny [61]. The molecular mechanisms underlying the differences in lipid metabolism between *lepa*^−/−^ and *lepb*^−/−^ zebrafish were further explored, and it was found that *lepb* mainly promotes lipolytic metabolism, whereas *lepa* mainly promotes anabolic lipid effects.

In summary, purebred mutant *lepa* and *lepb* zebrafish lines were successfully constructed by CRISPR/Cas9 gene editing in this study. The role of leptin in feeding growth, as well as in glycolipid metabolism, was explored in zebrafish fed a high-glucose diet. The anorectic effects of *lepa* and *lepb* in zebrafish were confirmed to be similar to those in mammals. *Lepa* plays a major role in glucose metabolism, favoring the promotion of glucose utilization and lipogenesis, whereas *lepb* mainly promotes lipolytic oxidation and regulates protein production but plays a minor role in glucose metabolism.

## 4. Materials and Methods

### 4.1. Acquisition of Mutants

The zebrafish used in the experiment were raised in an indoor recirculating water culture system at the Mandarin Fish Research Center of Huazhong Agricultural University (Wuhan, China). The temperature was maintained at approximately 26–28 °C year-round under a cycle of 14 h light and 10 h darkness. The ethics committee approval number for this experiment is HZAUFI-2020-0038. Wild-type zebrafish embryos were gene-edited using CRISPR/Cas9 technology, and knockout targets were designed on the second exon of *lepa* and *lepb* using an online tool (https://cctop.cos.uni-heidelberg.de/ (accessed on 22 October 2024)). pMD19-T plasmid was used as a template for PCR amplification, purified, and recovered, then transcribed in vitro with a TranscriptionAid T7 High Yield Transcription kit (Thermo Scientific, Waltham, MA, USA). sgRNA was recovered by purification with lithium chloride precipitation at the end of in vitro transcription. Equal volumes of 500 ng/µL Cas9 and 80 ng/µL sgRNA were mixed and injected into single-cell zebrafish embryos via microinjection. The mutated target fragment was detected by PCR amplification, and if a double peak appeared at the position of the sgRNA target sequence as a result of sequencing, the mutant F0 generation was raised to adulthood. The primers used for PCR detection of *lepa* and *lepb* are shown in Table S1. Mutant F0 parents were paired with wild-type fish to obtain the F1 generation. The F1 generation was raised with clipped caudal fins, and based on the sequencing results, males and females of the same mutation type were mated to obtain F2 pure *lepa*^−/−^ and *lepb*^−/−^ zebrafish haploids.

### 4.2. High-Glucose Diet Feeding

The high-glucose feed was made according to the experimental requirements and formulated based on the amino acid profile of zebrafish dorsal muscle. The formulation and composition of the high-carbohydrate diet are shown in Table S2. Crystalline L-amino acid premix was added to the experimental feed [62]. Wild-type and mutant zebrafish were fed *Artemia nauplii* reared to 60 days post fertilization (dpf). Males of uniform size for each genotype were randomly selected, including wild-type zebrafish, *lepa*^−/−^ zebrafish, and *lepb*^−/−^ zebrafish. Three parallel tanks were set up for each group, and 20 zebrafish were selected from each tank. During the 60-day experiment, fish were fed a high-glucose diet three times per day (08:30, 12:30, and 16:30) to apparent satiety.

### 4.3. Analysis of Growth Indices and Body Composition of Zebrafish

The weight and length of zebrafish were measured and recorded before and after they were fed a high-glucose diet. At the end of the high-glucose diet period, the liver and visceral mass were weighed separately. Mesenteric mucosal fat was collected to calculate the VSI, HSI, and MFI. The moisture content was determined using the drying method at 105 °C (GB/T5009.3-2016) [63]. The ash content of the sample was determined by constant temperature incineration at 550 °C (GB/T5009.4-2016) [64]. The protein content of the sample was determined by Kjeldahl nitrogen determination (GB/T5009.5-2016) [65], and the crude fat content was determined by the Soxhlet extraction method (GB/T5009.6-2016) [66].

### 4.4. Sample and Biochemical Analyses

After 60 d on a high-glucose diet, all the fish were anaesthetized with tricaine methanesulfonate (MS-222). The caudal fin was severed with scissors, and the whole blood was collected from the wound with a pipette tip treated with sodium heparin solution. The plasma was separated from the fish blood by refrigerated centrifuge (4 °C, 1500× *g*, 15 min).

Muscle and liver tissue were randomly taken from 6 fish of each genotype; the tissues from each group of fish were mixed together and divided equally into 3 portions, weighed, and packed in 2 mL test tubes. Glucose, triglyceride (TG), glycogen, protein, and total cholesterol levels were measured in plasma, liver, and muscle using commercial kits (Nanjing Jiancheng Bioengineering Institute, Nanjing, China). [Glucose Assay Kit, Triglyceride Assay Kit, Liver/Muscle Glycogen Assay Kit, The Total Protein Assay Kit and Total Cholesterol Assay Kit].

### 4.5. Statistics on Food Intake

After the high-glucose diet experiment, 18 fish of each genotype were randomly selected and weighed after starvation for 24 h, and the food intake of each type was measured. Pre-weighed feed was placed in each tank. After 2 h, the remaining feed from each tank was carefully sucked up with a straw, oven-dried, and weighed.

### 4.6. Quantitative Real-Time PCR

At the end of the high-glucose diet experiment, liver and muscle tissue samples were randomly taken from 12 fish of each genotype, and total RNA of the samples was extracted using TRIzol Reagent (Takara, Tokyo, Japan). cDNA synthesis was carried out using a Reverse Transcription Kit (Vazyme, Nanjing, China), and samples were stored at −20 °C for later use. Zebrafish-specific primers were designed using Primer Premier 6.0 software (Table S1), and quantitative real-time PCR was used to detect gene expression. The RT-PCR experimental system is described as follows. Each reaction mixture (20 μL) contained 1 μL cDNA template, 10 μL SYBR (Vazyme Nanjing, China), 0.4 μL of each primer, and 8.2 μL ddH_2_O. The cycling parameters were 95 °C for 30 s, 40 cycles at 95 °C for 10 s, 58 °C for 30 s, and a melting curve ranging from 65 °C to 95 °C (gradually increasing 0.5 °C s^−^^1^), with data acquired every 6 s.

### 4.7. Histological Analysis

Dorsal muscle and liver tissue samples from each genotype of zebrafish (*n* = 3) were randomly taken and fixed overnight by adding 4% paraformaldehyde (PFA); then, frozen sections of muscle and liver tissue with a thickness of 4 μm were made. The frozen sections of liver and muscle tissue were stained with Oil Red O and Alcian Blue-Phosphoric Acid Schiff (AB-PAS), respectively, to observe the changes in fat and glycogen contents of the samples.

### 4.8. Data Analysis

All data collected this experiment were expressed as the mean ± standard error (mean ± S.E.M.) and analyzed using IBM SPSS Statistics 25 software. The normality of the data was first tested by the Shapiro–Wilk test. A one-Sample *T* test was used to exclude sample data that deviated from the overall mean, and an independent *T* test was used to compare two groups of data, with *p* < 0.05 indicating a significant difference. Comparisons between multiple datasets were performed using one-way analysis of variance (ANOVA), and Duncan’s multiple range test was used for significant differences, with *p* < 0.05 indicating statistical significance.

## Figures and Tables

**Table 1:** Growth performance and whole-body proximate composition of three genotypes of zebrafish fed a high-glucose diet for 60 days.

Variable	Genotype		
	*wt*	*Lepa* ^−/−^	*Lepb* ^−/−^
Initial weight (IW), g	0.2136 ± 0.0023	0.2118 ± 0.0031	0.2111 ± 0.0035
Final weight (FW), g	0.4588 ± 0.0028 ^a^	0.5020 ± 0.0022 ^b^	0.4969 ± 0.0013 ^b^
Weight-gain rate (WGR) %	1.2613 ± 1.408 ^a^	1.3718 ±3.406 ^b^	1.3551 ±4.015 ^b^
Viscera-somatic index (VSI), %	8.44± 0.167	7.97± 0.129	8.73± 0.120
Hepato-somatic index (HSI), %	2.30 ± 0.0017	2.07± 0.0012	2.42± 0.0012
Mesenteric fat index (MFI), %	0.84 ± 0.013 ^b^	0.72 ± 0.014 ^a^	1.26 ± 0.018 ^c^
Moisture, %	68.53 ± 0.496	68.87 ± 0.392	69.52 ± 0.386
Crude protein, %DM	46.91± 0.750 ^a^	49.18± 0.521 ^a^	50.74± 0.180 ^b^
Crude fat, %DM	28.73 ± 0.692 ^b^	25.75 ± 0.649 ^a^	36.29 ± 0.401 ^c^
Ash, %DM	10.00	9.90	10.24

Note: The data in the table are the average values and standard errors of mixed samples of each group (*n* = 3). Different letters of (a, b, and c) indicate the significance of the three genotypes of zebrafish. Those with different letters are significant (*p* < 0.05), while those without letters are not significant (*p* > 0.05).

## Data Availability

All data are available from the corresponding author by request.

## Supplementary Materials

The following supporting information can be downloaded at: https://www.mdpi.com/article/10.3390/ijms252111647/s1.
