# Supplementary material for: Study on the Function of Leptin Nutrient Acquisition and Energy Metabolism of Zebrafish (Danio rerio)
Source: Int J Mol Sci. 2024 Oct 30;25(21):11647. doi: 10.3390/ijms252111647 (PMC11546987; doi:10.3390/ijms252111647)
Supplement: Supplementary file 1 [file ijms-25-11647-s001.zip › ijms-3273869-supplementary.pdf]

## Table S1. Primers used in the experiment

| Gene name | Primer | sequences (5′-3′) | Annealing temp (℃) |
|---|---|---|---|
| *leptina* | *lepa*-F | AATGTCAAACTGCAGGCAAAG | 55 |
| | *lepa*-R | TTTCAGCTGGTCCAGATTATCG | |
| *leptinb* | *lepb*-F | TCCATCAGCTTAGTTCTTGATT | 55 |
| | *lepb*-R | CCTTGCATGTGCCATTGT | |
| *npy* | *npy*-F | CTGTGATGTCCATGTGTCCTTCTG | 58 |
| | *npy*-R | GAGCCTAAAGAGCGCACATTGA | |
| *agrp* | *agrp*-F | AGACCTTGAAGCCTATGATGAG | 56 |
| | *agrp*-R | GCCTTAAAGAAGCGGCAGTA | |
| *pomc* | *pomc*-F | CAGAGTCTGAGCTTGGGTTTGCTT | 58 |
| | *pomc*-R | ACTTTTACCGGTCTGCGTTTGC | |
| *cart* | *cart*-F | GTAAAGCCCCGAGGAGCTGCTTT | 58 |
| | *cart*-R | CATACAGGCTGCAGAAATGGACTT | |
| *gys2* | *gys2*-F | GTGCGATGCGACTATCCAGA | 60 |
| | *gys2*-R | TTCACCCCATTCGTCCACAG | |
| *gp* | *gp*-F | AGAAGCCGGAGAGGAAAACC | 60 |
| | *gp*-R | TCTCAGGCTGTTTCGGTGAA | |
| *gck* | *gck*-F | CACCGCTGACCTGCTATGAT | 58 |
| | *gck*-R | AGTCGGCCACTTCACATACG | |
| *pk* | *pk*-F | TCCTGGAGCATCTGTGTCTG | 58 |
| | *pk*-R | GTCTGGCGATGTTCATTCCT | |
| *g6pca.1* | *g6pca.1*-F | GCTGCACCATACGAGATGGA | 58 |
| | *g6pca.1R* | TCACCAAACAGCACCCACTT | |
| *pck1* | *pck1*-F | CGCGTACTGGAGTGGATGTT | 58 |
| | *pck1*-R | GTGTGTTGCGTGTCTTCAGC | |
| *cpt1b* | *cpt1b*-F | TGAGACGGATTCTTTCCGCT | 56 |
| | *cpt1b*-R | TTCGCTAGGCTTGTTACTTGC | |
| *fas* | *fas*-F | TCCAAGAGTTCAAACACGGT | 60 |
| | *fas*-R | TGAGTGACACCACAACAG | |
| *acc* | *acc*-F | ATGGCAGAGCAAGACTCCAC | 58 |
| | *acc*-R | CCTCTGCAGGTCGATACGTC | |
| *pparα* | *ppar*-F | CTGGTGTACGATCACTGCGA | 54 |
| | *ppar*-R | TGTGGTTCACGTCACTGGAG | |
| *pparγ* | *pparγ*-F | CTGGTGTACGATCACTGCGA | 58 |
| | *pparγ*-R | TGTGGTTCACGTCACTGGAG | |
| *β-actin* | *β-actin*-F | CGAGCAGGAGATGGGAACC | 56 |
| | *β-actin*-R | CAACGGAAACGCTCATTGC | |

**Table S2. The formulation and composition of the high-glucose diet**

| Ingredients | Content, % |
|---|---|
| Casein | 33.00 |
| Gelatin | 2.00 |
| Dextrin | 24.00 |
| Corn starch | 24.00 |
| Soybean oil | 8.00 |
| [1]Vitamin premix | 0.90 |
| Choline chloride | 0.10 |
| [2]Mineral premix | 1.00 |
| Monocalcium phosphate | 2.50 |
| Crystalline amino acid | |
| L-Ala | 1.00 |
| L-Arg | 1.00 |
| L-Asp | 1.00 |
| L-Gly | 1.00 |
| L-Lys | 0.50 |
| Proximate composition | |
| Moisture, % | 13.35 |
| Crude protein, %DM | 36.35 |
| Crude fat, %DM | 5.50 |
| [3]Carbohydrate, %DM | 41.50 |
| Ash, %DM | 3.30 |

1.Vitamin premix (mg/kgdiet): vitamin D3, 0.05; vitamin A1, 6.9; vitamin K3, 25; vitamin E, 100; vitamin B1 (thiamin), 30; vitamin B2 (riboflavin), 30; vitamin B6, 20; vitamin B12, 0.1; nicotinic acid, 200; folic acid, 15; ascorbic acid, 1000; inositol, 500; vitamin H, 3; pantothenic acid calcium, 100 (Gao Long Dietary Company).

2.Mineral premix (mg/kg diet): CoSO4.H2O, 0.65; CuSO4.5H2O, 9; FeSO4.7H2O, 8.34; KI, 0.5; MnSO4. H2O, 22.85; Na2SeO3, 0.01; ZnSO4.7H2O, 14.3; NaCl, 400; CaCO3, 1860; MgSO4, 240 (Gao Long Dietary Company).

3.Carbohydrate [%dry matter] =100-(crude protein-crude fat -crude ash)
